# Doxorubicin Anticancer Drug Monitoring by ds-DNA-Based Electrochemical Biosensor in Clinical Samples

**DOI:** 10.3390/mi12070808

**Published:** 2021-07-09

**Authors:** Firouzeh Hassani Moghadam, Mohammad A. Taher, Hassan Karimi-Maleh

**Affiliations:** 1 Department of Chemistry, Shahid Bahonar University of Kerman, Kerman 7616913439, Iran; fhassanimoghaddam@gmail.com; 2 Laboratory of Nanotechnology, Department of Chemical Engineering and Energy, Quchan University of Technology, Quchan 9477177870, Iran

**Keywords:** docking investigation, doxorubicin, Sensor, modified biosensor

## Abstract

In this research, glassy carbon electrode (GCE) amplified with single-wall carbon nanotubes (SWCNTs) and ds-DNA was fabricated and utilized for voltammetric sensing of doxorubicin with a low detection limit. In this technique, the reduction in guanine signal of ds-DNA in the presence of doxorubicin (DOX) was chosen as an analytical factor. The molecular docking study revealed that the doxorubicin drug interacted with DNA through intercalation mode, which was in agreement with obtained experimental results. The DOX detection performance of ds-DNA/SWCNTs/GCE was assessed at a concentration range of 1.0 nM–20.0 µM. The detection limit was found to be 0.6 nM that was comparable and even better (in many cases) than that of previous electrochemical reported sensors. In the final step, the ds-DNA/SWCNTs/GCE showed powerful ability for determination of the DOX in injection samples with acceptable recovery data.

## 1. Introduction

Cancer is regarded as one of the most important diseases in the world as well as the primary cause of mortality for many individuals [1,2]. The use of chemotherapy and anticancer drugs is the best and most important way to deal with this deadly disease [3,4]. Breast cancer is one of the most important and prevalent kinds of cancer that has been diagnosed [5]. Doxorubicin is one of the most important and extensively used drugs with an appropriate response for the treatment of breast cancer, which has been used for this purpose for many years [6,7]. This drug is one of the initial options of doctors in the treatment of breast cancer [8,9]. However, due to the drug’s adverse effects, its regulated dosage is essential in chemotherapy [10]. Therefore, its continuous measurement in biological environments following treatment is crucial and required [11,12,13,14,15]. Numerous methods have been used for this purpose, such as high-performance liquid chromatography (HPLC), spectroscopic methods, and electrochemical sensors [11,16,17,18,19].

Electrochemical strategies showed many advantages for sensing of biological, anticancer drugs and a wide range of electroactive materials due to easy operation, fast response, high sensitivity, and portable ability [20,21,22,23,24,25]. Furthermore, the electrochemical sensors are considered to be more efficient and sensitive than other methods due to the opportunity to be boosted using various modifiers [26,27,28,29]. As a result of these considerations, numerous teams have reported electrochemically modified sensors for DOX monitoring [15,30,31,32]. However, the low selectivity issue is still one of the most important obstacles of electrochemical sensors for the detection of DOX. Due to the specific reaction, biological recognition elements, such as DNA, can be used to overcome this issue [33,34].

The intercalation of DOX with ds-DNA inhibits macromolecular biosynthesis, thereby it helps in the progression of topoisomerase II, an enzyme that relaxes supercoils in DNA for transcription [35]. On the other hand, intercalation between ds-DNA bases and doxorubicin allows the formation of a highly sensitive and selective strategy for electrochemical monitoring of DOX, which has also been confirmed by different groups [36].

**Scheme 1:**
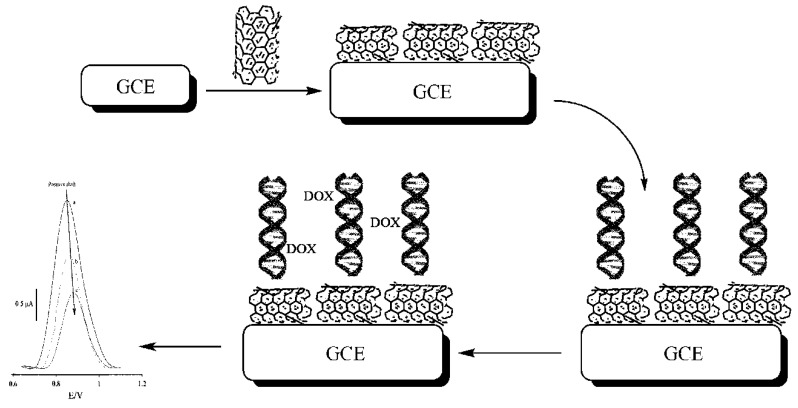
The schematic illustration of the present work.

Nanotechnology is now considered to be an important part of engineering processes in the world [37,38,39,40,41,42]. The unique properties of nanomaterials have led to their widespread application in industry and the design of engineering systems and have led to significant changes in various scientific fields [43,44,45,46,47,48,49]. SWCNTs, meanwhile, have been surprisingly improved as a one-dimensional nanomaterial with high electrical conductivity in improving the properties of electrochemical sensors [50]. Bearing all points in mind, herein, it was aimed to design an SWCNTs-modified sensor to be utilized in DOX analysis. The schematic illustration of the proposed work was given in Scheme 1. The findings revealed that the ds-DNA/SWCNTs/GCE showed good selectivity and high sensitivity for sensing of doxorubicin in pharmaceutical samples.

## 2. Materials and Methods

### 2.1. Materials and Instrument

Calf Thymus DNA (double-stranded), boric acid, phosphoric acid, Tris hydrochloride, acetic acid, doxorubicin hydrochloride, and SWCNTs-COOH (>90% carbon basis, D × L 4–5 nm × 0.5–1.5 μm) were purchased from Across and Sigma-Aldrich Companies. Stock solution of doxorubicin hydrochloride (0.001 M) was prepared by dissolving 0.0058 g doxorubicin hydrochloride in 10 mL buffer solution. An Ivium-Vertex machine connected to the three-electrode experimental setup consisting of Ag/AgCl/KCl_sat_ reference, Pt wire (Azar Electrode, Tehran, Iran) and ds-DNA/SWCNTs/GCE were employed for differential pulse voltammetric (DPV) (pulse height of 50 mV and a pulse width of 5 mV) analysis.

### 2.2. Preparation of ds-DNA/SWCNTs/GCE

GCE (A = 0.314 cm^2^, Azar Electrode, Iran) was polished via alumina powder and washed by distilled water solution. Subsequently, 10 mL of SWCNTs-COOH suspension was dropped at the surface of clean GCE and dried for 10 min. The ds-DNA was prepared into acetate buffer (0.5 M, pH 4.8). Afterwards, 10 µL of ds-DNA solution (50 mg/L) was dropped at the surface of SWCNTs/GCE.

### 2.3. Preparation of Clinical Samples

Injection samples of doxorubicin (2 mg/mL) and dextrose saline were purchased from a local pharmacy and used for clinical samples analysis without any pretreatment. Samples were diluted in tris-HCl buffer solution pH 7.4 and transferred to electrochemical cell for clinical sample analysis by standard addition strategy. Different concentrations of doxorubicin were spiked in dextrose saline and used for real sample analysis.

### 2.4. Experimental Procedure

Doxorubicin solution was prepared in tris-HCl buffer solution pH 7.4. Intercalation of doxorubicin and ds-DNA was checked in tris-HCl buffer solution at pH 7.4 for 20 min. Finally, the electrode was transferred into another electrochemical cell and relative voltammograms were recorded in an acetate solution (0.5 M, pH 4.8). All of the experiments were repeated four times. Electrochemical impedance spectroscopy (EIS) was employed in the frequency range of 100 kHz to 1.0 Hz.

### 2.5. Molecular Docking

Molecular docking was conducted to predict the favorable DOX-DNA complexes using AutoDock Vina [51] and AutoDockTools (ADT) V.1.5.6 [52]. 3D structures of DNA dodecamer (PDB ID: 1BNA) and DNA hexamer containing an intercalation gap (PDB ID:1Z3F) were extracted from RCSB protein data bank [53]. Moreover, the 3D structure of DOX drug was downloaded from PubChem database [4] (CID 31703). The partial atomic charges for both DNA receptor and DOX were assigned using the Kollman united atom [54] and Gasteiger–Marsili [55] methods, respectively. The parameters of DOX-DNA complexes were prepared using ADT, which were denoted in the PDBQT file. The grid maps of dimensions 72 × 72 × 122 Å (80 × 68 × 82 Å) with a grid-point spacing of 0.375 Å were created around the center of 1BNA (1Z3F) so that the whole structure was included. In addition, the grid box centers for x, y and z coordinates of 1BNA receptor (1Z3F) are 14.779 Å, 20.976 Å, and 8.804 Å (2.303 Å, 15.703 Å, and 37.592 Å). During the docking process, DOX molecule was allowed to move freely, whereas the receptor molecule was kept rigid and fixed. The structures of DOX-DNA docked models were visualized using PyMol software [56].

## 3. Results and Discussion

### 3.1. Modification of Electrode Surface

The EIS technique was used to examine the modification of the bare electrode by SWCNTs. The signal of 1.0 mM [Fe(CN)6]^3−,4−^ + 1.0 mM KCl was selected as a reference signal for this goal. The charge transfer resistances were obtained as 2.1 KΩ, 1.4 KΩ, and 1.85 KΩ for GCE and GCE modified with SWCNTs and GCE modified with SWCNTs and ds-DNA, respectively, confirming the higher conductivity of SWCNTs at the surface of GCE.

### 3.2. Electrochemical Sensing of Doxorubicin

**Figure 1:**
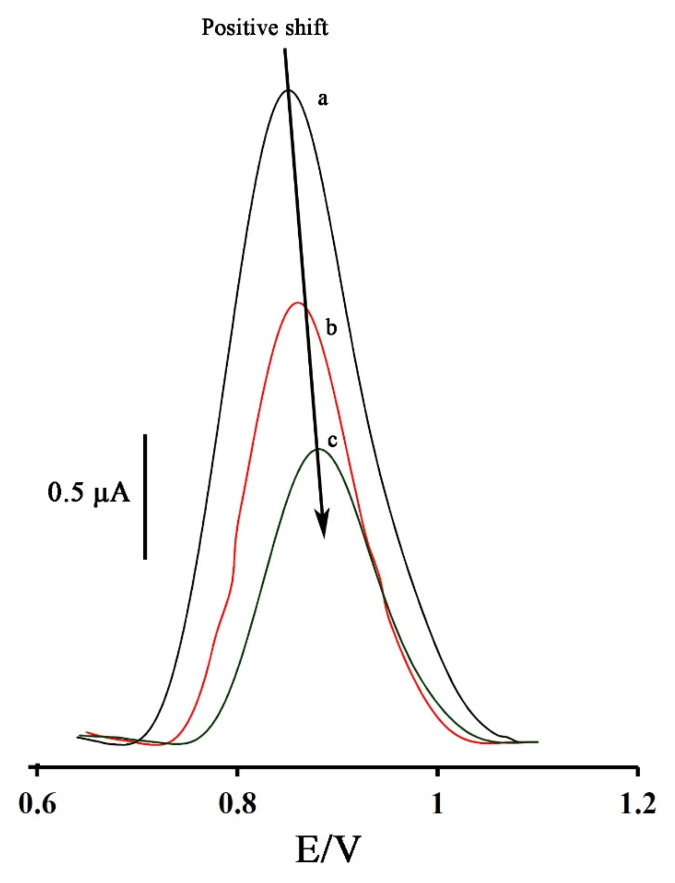
DP voltammograms of ds-DNA/SWCNTs/GCE in the absence (**a**) and in the presence of 7.0 µM (**b**) and 14.0 µM (**c**) doxorubicin, respectively.

The ds-DNA/SWCNTs/GCE voltammograms in the absence (curve a) of doxorubicin and in the presence of 7.0 and 14.0 µM doxorubicin (curves b and c) are depicted in Figure 1. It was clear that the currents of guanine base were significantly dependent on the concentration of doxorubicin in the solution. With increasing doxorubicin concentration from 0.0 µM to 7.0 and 14.0 µM in the solution, the oxidation signal of guanine reduced from 2.6 µA to 1.74 µA and 1.17 µA, respectively. In addition, a positive shift could be observed in guanine signal after the addition of doxorubicin, confirming the intercalation between ds-DNA at the surface of the sensor and doxorubicin.

### 3.3. Optimization of ds-DNA Concentration

**Figure 2:**
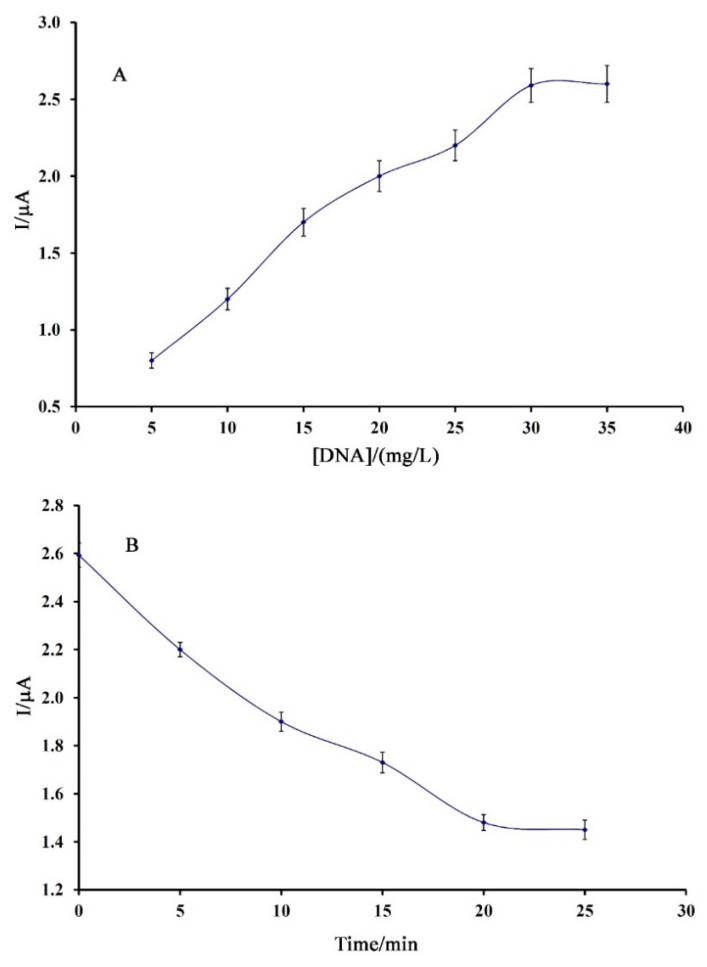
(**A**) Current-concentration plot of ds-DNA/SWCNTs/GCE fabricated with different initial concentrations of ds-DNA. (**B**) Current-time plot of ds-DNA/SWCNTs/GCE (ds-DNA 30.0 mg/L) in the presence of 7.0 µM doxorubicin.

ds-DNA concentration is one of the most important factors in DNA-based biosensor applications. Therefore, the ds-DNA concentration was optimized before linear dynamic range investigation and clinical sample analysis. The differential pulse voltammograms of ds-DNA/SWCNTs/GCE prepared with different initial concentrations of ds-DNA was also recorded at the same experimental conditions (not shown). The current concentration diagram relative to DP voltammograms is given in Figure 2A. As can be seen, with increasing the initial concentration of ds-DNA, the oxidation current of guanine base increased and then reached a stable condition at the concentration of 30 mg/L. After this value, the oxidation current of dsDNA/SWCNTs/GCE did not change with increasing ds-DNA, indicating that the electrode surface was saturated. Therefore, 30 mg/L ds-DNA was selected as the optimum concentration for monitoring ds-DNA/SWCNTs/GCE to sensing of doxorubicin.

In addition, intercalation time between 30.0 mg/L ds-DNA and 7.0 µM anticancer drug was optimized as one of the main factors in the ability of the sensor for trace-level analysis of doxorubicin in the period time 0–25 min. As can be observed, with increasing the time until 20 min, the oxidation current of guanine reduced and then remained stable (Figure 2B). This referred that 20 min was sufficient for intercalation between analytes and ds-DNA.

### 3.4. Linear Dynamic Range and Limit of Detection

**Figure 3:**
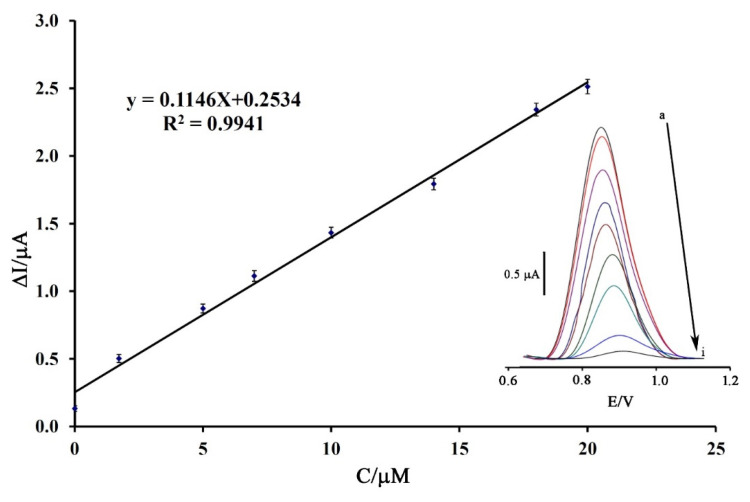
Current concentration plot of ds-DNA/SWCNTs/GCE in the presence of 1.0 nM–20.0 µM doxorubicin at the optimum condition (*n* = 4). (Inset) DP voltammogram of ds-DNA/SWCNTs/GCE in the presence of 1.0 nM–20.0 µM (a–i; 0.0, 0.001, 1.72, 5.0, 7.0, 10.0, 14.0, 18.0, and 20.0 µM) doxorubicin at the optimum condition.

DP voltammograms of ds-DNA/SWCNTs/GCE in the presence different concentrations of doxorubicin were recorded and represented in the inset of Figure 3. As can be seen, the oxidation current of the guanine base displayed a linear dynamic range in the concentration range 1.0 nM–20.0 µM for sensing of doxorubicin at the optimum conditions (Figure 3). Moreover, the findings indicated that the ds-DNA/SWCNTs/GCE could be successfully utilized for the determination of doxorubicin with a limit of detection (LOD) of 0.6 nM (Y_LOD_ = 3 S_b_/m; S_b_ is the standard deviation of blank and m is the slope of linear dynamic range). The linear dynamic range and limit of detection of the proposed biosensor were comparable with previous electrochemical suggested sensors for monitoring of DOX (see Table 1).

### 3.5. Analysis of the Clinical Samples

The ability of ds-DNA/SWCNTs/GCE for sensing of doxorubicin was checked in various clinical samples, including injection and dextrose saline. Dextrose saline was selected as a complex matrix that usually has injections added in it. The standard addition results for the determination of doxorubicin were tabulated in Table 2. The recovery range of 97.2–104.9% offered the high-performance ability of ds-DNA/SWCNTs/GCE, implying the potential for utilization as a new biosensor for the determination of doxorubicin in clinical samples.

### 3.6. Molecular Docking Investigation

**Figure 4:**
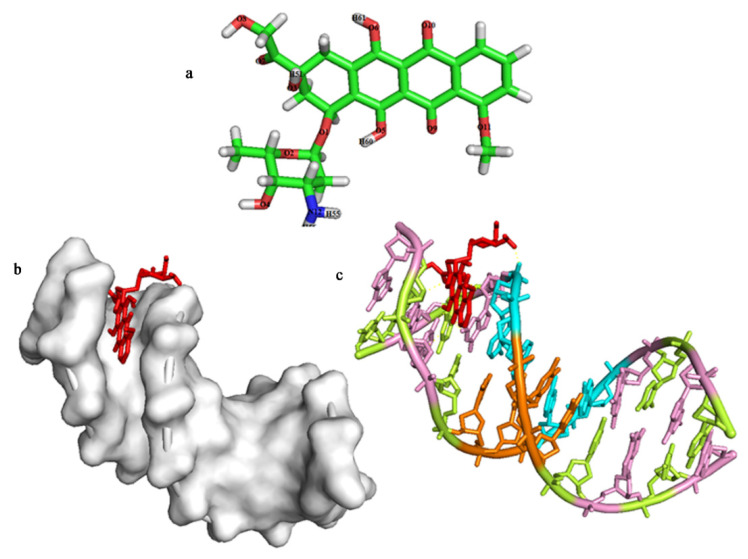
(**a**) Chemical structure of Doxorubicin drug with the numbering atoms discussed in the text, (**b**) surface view of Doxorubicin binding in minor groove of duplex of sequence d(CGCGAATTCGCG)2 and (**c**) structure of Doxorubicin-dodecamer DNA (1BNA) docked complex (color codes: deoxyadenosine (DA): cyan, deoxcytosine (DC): limon, deoxyguanosine (DG): pink, deoxythymine: orange and Doxorubicin: red).

**Figure 5:**
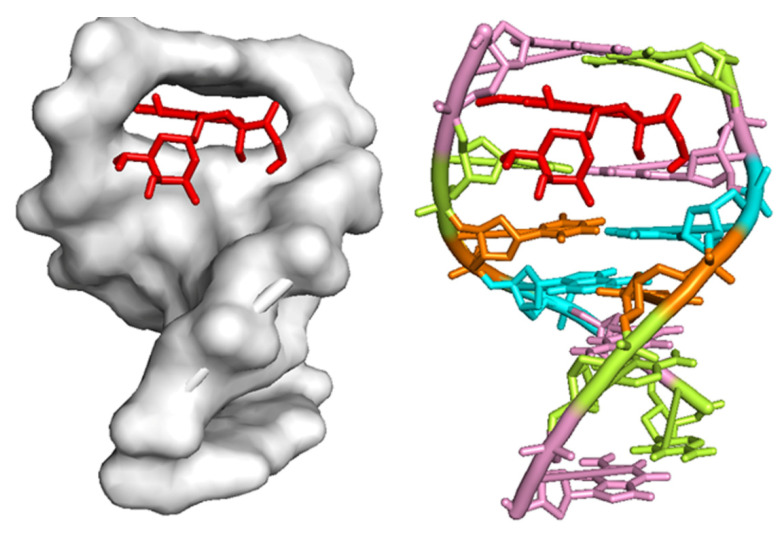
The intercalation binding mode of Doxorubicin into DNA hexamer (1Z3F) (**right**) and surface view of Doxorubicin-hexamer docked complex (**left**).

**Figure 6:**
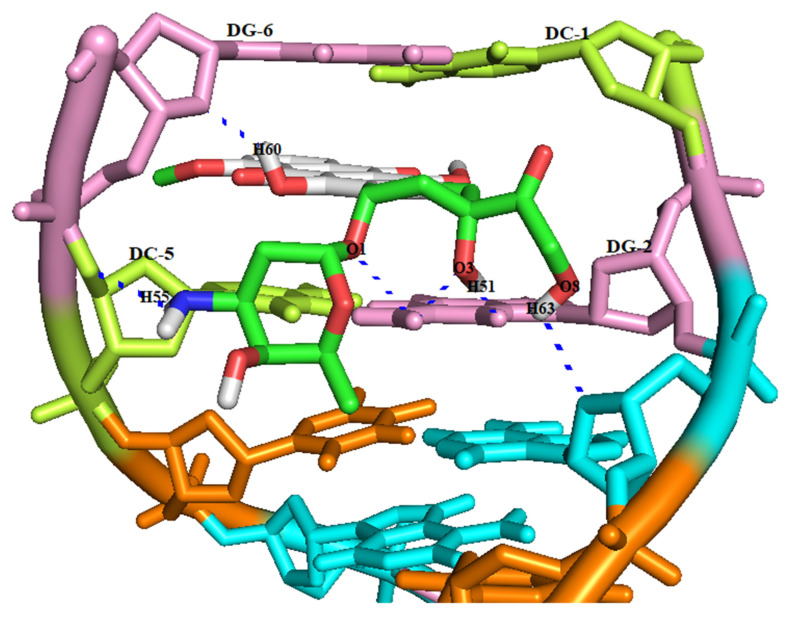
The hydrogen bonding interaction of doxorubicin drug to adjacent cytosine and guanine residues of DNA hexamer.

The binding affinity of doxorubicin (DOX) drug inside the DNA receptor was theoretically assessed in two manners: Groove interaction and intercalation. For those, DNA structures with base pairs sequences dodecamer d(CGCGAATTCGCG)2 (PDB ID: 1BNA) and hexamer d(CGATCG)2 (PDB ID: 1Z3F) were used. The best conformations of DOX-DNA docked models with the lowest binding affinity are visualized in Figure 4, Figure 5 and Figure 6.

#### 3.6.1. Minor Groove Docking Study

Molecular docking revealed that the binding mode of DOX drug to DNA dodecamer was via minor groove manner with the binding affinity of −9.1 kcal/mol (see Figure 4).

As shown in this figure, doxorubicin drug was located in the vicinity of cytosine and guanine base pairs of DNA in such a way DOX forms O…H-O and O…H-N conventional hydrogen bonds (HBs) with DNA. The mentioned interactions were as follows: (I) The oxygen (O4′) atom of 16th guanine of DNA interacts with the hydrogen (H51) atom of drug molecule with a hydrogen bond distance of 2.4 Å and a bond angle of about 133.1°; (II) the hydrogen (H56) atom of NH2 group of drug interacts with (O)P1 of deoxyadenosine 17 (DA-17) with a bond length of 3.7 Å and a bond angle of about 130.3°. The oxygen atom (O6) of hydroxyl termination of drug molecule was 2.8 Å from the nitrogen atoms (N2) of DG-16 chain B and DG-10 chain A. The nitrogen (N2) atom of deoxyguanosine 14 (DG-14) chain B was doubly bonded to oxygen atoms of drug molecule with N2…O8 and N2…O7 distances of 2.8 and 3.1 Å, respectively. Moreover, the oxygen (O10) atom of drug molecule was bonded with N2 atom of DG-16 chain B and O4′ atom of DG-10 chain A with bond distances of 3.3 and 3.5 Å, respectively.

#### 3.6.2. Intercalation Docking

It was found that doxorubicin was stabilized in the intercalating site of the hexamer of DNA in which drug was surrounded by nitrogenous cytosine (DC-1 and DC-5) and guanine (DG-2 and DG-6) base pairs of DNA with a binding affinity of −9.6 kcal/mol (see Figure 5). In addition, it was observed that the hydrogen and oxygen atoms of DOX drug were participated as the donor and acceptor moieties to form three O…H-N and two O…H-O conventional HBs with DNA receptor (see Figure 6). The preferential intercalative binding poses of DOX drug were approved by a higher negative value of binding affinity of the DOX with DNA hexamer in comparison with DNA dodecamer. The hydrogen atom (H21) of deoxyguanosine 2 (DG-2) chain B was doubly bonded with O1 and O3 atoms of DOX molecule, those are, O1…H21-N2 and O3…H21-N2 (rO1…H21-N2 = 2.8 Å with <NHO = 103.0° and rO3…H21-N2 = 2.2 Å with <NHO = 132.4°).

Moreover, hydrogen atom of hydroxyl termination of drug molecule as the proton donor interacted with O4′ atom of deoxyribose sugar moiety linked to adenine (DA-3 of chain B) as a proton acceptor with a distance of 2.7 Å and a bond angle of about 101.8°. The oxygen atom of deoxyribose sugar moiety of 6th guanine DNA interacted with hydrogen atom of drug molecule with O4′…H60-O5 hydrogen bond distance of 2.2 Å and a bond angle of about 143.4°. Furthermore, the hydrogen atom (H55) of NH2 terminal group of drug molecule was bonded to the oxygen atom (O3′) of deoxycytosine (DC-5 of chain A) with a bond length of 2.6 Å and a bond angle of about 117.3°. In addition, the intermolecular interaction between hydrogen atom of hydroxyl group of DOX drug and hydrogen atom of DG-2 chain B DNA with H51…H3 bond length of 1.5 Å was observed.

## 4. Conclusions

In this study, a highly sensitive and powerful electroanalytical biosensor was fabricated and utilized for sensing of doxorubicin. ds-DNA/SWCNTs/GCE offered an intercalation reaction between ds-DNA and doxorubicin and used as analytical approach for sensing of doxorubicin with a detection limit of 0.6 nM. The presence of single-wall carbon nanotubes at the surface of GCE boosted the sensitivity of the sensor for trace-level analysis. Moreover, the docking investigation confirmed the satisfactory intercalation between adenine and guanine bases of ds-DNA and validated the experimental part.

## Figures and Tables

**Table 1:** The comparison of the reported analytical parameters of similar electrochemical sensors for monitoring DOX.

Electrode	Mediator	LDR (µM)	LOD (µM)	Ref.
Glassy Carbon	Graphene quantum dot	0.018–3.600	0.016	[57]
Carbon paste	ZnO nanoparticle/1-butyl-3-methylimidazolium tetrafluoroborate	0.07–500	0.009	[11]
Hanging mercury drop electrode	---	0.5–10.0	0.1	[58]
Glassy Carbon	Cyclodextrin-graphene hybrid nanosheets	0.01–0.2	0.0001	[59]
Screen-printed	bird nest-like nano-structured NiCo_2_O_4_	0.01–600.0 μM	0.0094	[60]
Glassy Carbon	ds-DNA/SWCNTs	0.001–20.0	0.0006	This work

**Table 2:** The clinical samples analysis data for determination of doxorubicin using ds-DNA/SWCNTs/GCE as a new biosensor (*n* = 4).

Sample	Doxorubicin Added (µM)	Doxorubicin Expected (µM)	Doxorubicin Founded (µM)	Recovery% (Doxorubicin Founded/Doxorubicin Expected) * 100
Injection	-	1.00	1.03 ± 0.05	103
-	9.00	10.00	9.72 ± 0.53	97.2
dextrose saline	-	-	<Limit of detection	-
-	10.00	10.00	9.82 ± 0.53	98.2
-	15.00	15.00	15.74 ± 0.86	104.9

## References

[1] Forouzanfar S., Alam F., Pala N., Wang C. (2020). A Review of Electrochemical Aptasensors for Label-Free Cancer Diagnosis. J. Electrochem. Soc..

[2] Gale R.P., Phillips G.L., Lazarus H.M. (2021). New cancer therapies. Are haematopoietic cell transplants a dead duck?. Bone Marrow Transplant..

[3] Reiffers J., Huguet F., Stoppa A., Molina L., Marit G., Attal M., Gastaut J., Michallet M., Lepeu G., Broustet A. (1996). A prospective randomized trial of idarubicin vs. daunorubicin in combination chemotherapy for acute myelogenous leukemia of the age group 55 to 75. Leukemia.

[4] Coleridge S.L., Bryant A., Kehoe S., Morrison J. (2021). Chemotherapy versus surgery for initial treatment in advanced ovarian epithelial cancer. Cochrane Database Syst. Rev..

[5] Kabir M., Rahman M., Akter R., Behl T., Kaushik D., Mittal V., Pandey P., Akhtar M.F., Saleem A., Albadrani G.M. (2021). Potential role of curcumin and its nanoformulations to treat various types of cancers. Biomolecules.

[6] Singal P.K., Iliskovic N. (1998). Doxorubicin-induced cardiomyopathy. N. Engl. J. Med..

[7] Chen C., Lu L., Yan S., Yi H., Yao H., Wu D., He G., Tao X., Deng X. (2018). Autophagy and doxorubicin resistance in cancer. Anti Cancer Drugs.

[8] Lankelma J., Dekker H., Luque R.F., Luykx S., Hoekman K., Van Der Valk P., Van Diest P.J., Pinedo H.M. (1999). Doxorubicin gradients in human breast cancer. Clin. Cancer Res..

[9] Lovitt C.J., Shelper T.B., Avery V.M. (2018). Doxorubicin resistance in breast cancer cells is mediated by extracellular matrix proteins. BMC Cancer.

[10] Carvalho C., Santos R.X., Cardoso S., Correia S., Oliveira P.J., Santos M.S., Moreira P.I. (2009). Doxorubicin: The good, the bad and the ugly effect. Curr. Med. Chem..

[11] Alavi-Tabari S.A., Khalilzadeh M.A., Karimi-Maleh H. (2018). Simultaneous determination of doxorubicin and dasatinib as two breast anticancer drugs uses an amplified sensor with ionic liquid and ZnO nanoparticle. J. Electroanal. Chem..

[12] Fouladgar M. (2018). CuO-CNT nanocomposite/ionic liquid modified sensor as new breast anticancer approach for determination of doxorubicin and 5-fluorouracil drugs. J. Electrochem. Soc..

[13] El-Maghrabey M., Kishikawa N., Kamimura S., Ohyama K., Kuroda N. (2021). Design of a dual functionalized chemiluminescence ultrasensitive probe for quinones based on their redox cycle. Application to the determination of doxorubicin in lyophilized powder and human serum. Sens. Actuators B Chem..

[14] Yang M., Yan Y., Liu E., Hu X., Hao H., Fan J. (2021). Polyethyleneimine-functionalized carbon dots as a fluorescent probe for doxorubicin hydrochloride by an inner filter effect. Opt. Mater..

[15] Alarfaj N.A., El-Tohamy M.F. (2020). New Functionalized Polymeric Sensor Based NiO/MgO Nanocomposite for Potentiometric Determination of Doxorubicin Hydrochloride in Commercial Injections and Human Plasma. Polymers.

[16] Baurain R., Deprez-De Campeneere D., Trouet A. (1979). Determination of daunorubicin, doxorubicin and their fluorescent metabolites by high-pressure liquid chromatography: Plasma levels in DBA 2 mice. Cancer Chemother. Pharmacol..

[17] Sastry C.S., Rao J.S.L. (1996). Determination of doxorubicin hydrochloride by visible spectrophotometry. Talanta.

[18] Choi W.-G., Kim D.K., Shin Y., Park R., Cho Y.-Y., Lee J.Y., Kang H.C., Lee H.S. (2020). Liquid Chromatography–Tandem Mass Spectrometry for the Simultaneous Determination of Doxorubicin and its Metabolites Doxorubicinol, Doxorubicinone, Doxorubicinolone, and 7-Deoxydoxorubicinone in Mouse Plasma. Molecules.

[19] Mirzaei-Kalar Z., Yavari A., Jouyban A. (2020). Increasing DNA binding affinity of doxorubicin by loading on fe3o4 nanoparticles: A multi-spectroscopic study. Spectrochim. Acta Part A Mol. Biomol. Spectrosc..

[20] Karimi-Maleh H., Alizadeh M., Orooji Y., Karimi F., Baghayeri M., Rouhi J., Tajik S., Beitollahi H., Agarwal S., Gupta V.K. (2021). Guanine-based DNA biosensor amplified with Pt/SWCNTs nanocomposite as analytical tool for nanomolar determination of daunorubicin as an anticancer drug: A docking/experimental investigation. Ind. Eng. Chem. Res..

[21] Medetalibeyoğlu H., Beytur M., Manap S., Karaman C., Kardaş F., Akyıldırım O., Kotan G., Yüksek H., Atar N., Yola M.L. (2020). Molecular Imprinted Sensor Including Au Nanoparticles/Polyoxometalate/Two-Dimensional Hexagonal Boron Nitride Nanocomposite for Diazinon Recognition. ECS J. Solid State Sci. Technol..

[22] Karaman C., Karaman O., Yola B.B., Ulker İ., Atar N., Yola M.L. (2021). A novel electrochemical Aflatoxin B1 immunosensor based on gold nanoparticles decorated porous graphene nanoribbon and Ag nanocubes incorporated MoS2 nanosheets. New J. Chem..

[23] Böke C.P., Karaman O., Medetalibeyoglu H., Karaman C., Atar N., Yola M.L. (2020). A new approach for electrochemical detection of organochlorine compound lindane: Development of molecular imprinting polymer with polyoxometalate/carbon nitride nanotubes composite and validation. Microchem. J..

[24] Tajik S., Beitollahi H., Biparva P. (2018). Methyldopa electrochemical sensor based on a glassy carbon electrode modified with Cu/TiO2 nanocomposite. J. Serb. Chem. Soc..

[25] Tajik S., Beitollahi H. (2019). A sensitive chlorpromazine voltammetric sensor based on graphene oxide modified glassy carbon electrode. Anal. Bioanal. Chem. Res..

[26] Beitollahi H., Mahmoudi Moghaddam H., Tajik S. (2019). Voltammetric Determination of Bisphenol A in Water and Juice Using a Lanthanum (III)-Doped Cobalt (II, III) Nanocube Modified Carbon Screen-Printed Electrode. Anal. Lett..

[27] Beitollahi H., Safaei M., Tajik S. (2019). Different electrochemical sensors for determination of dopamine as neurotransmitter in mixed and clinical samples: A review. Anal. Bioanal. Chem. Res..

[28] Karimi-Maleh H., Orooji Y., Karimi F., Alizadeh M., Baghayeri M., Rouhi J., Tajik S., Beitollahi H., Agarwal S., Gupta V.K. (2021). A critical review on the use of potentiometric based biosensors for biomarkers detection. Biosens. Bioelectron..

[29] Karaman C., Karaman O., Atar N., Yola M.L. (2021). Electrochemical immunosensor development based on core-shell high-crystalline graphitic carbon nitride@ carbon dots and Cd 0.5 Zn 0.5 S/d-Ti 3 C 2 T x MXene composite for heart-type fatty acid–binding protein detection. Microchim. Acta.

[30] Chekin F., Myshin V., Ye R., Melinte S., Singh S.K., Kurungot S., Boukherroub R., Szunerits S. (2019). Graphene-modified electrodes for sensing doxorubicin hydrochloride in human plasma. Anal. Bioanal. Chem..

[31] Sharifi J., Fayazfar H. (2021). Highly sensitive determination of doxorubicin hydrochloride antitumor agent via a carbon nanotube/gold nanoparticle based nanocomposite biosensor. Bioelectrochemistry.

[32] Wang M., Lin J., Gong J., Ma M., Tang H., Liu J., Yan F. (2021). Rapid and sensitive determination of doxorubicin in human whole blood by vertically-ordered mesoporous silica film modified electrochemically pretreated glassy carbon electrodes. RSC Adv..

[33] Mahmoudi-Moghaddam H., Tajik S., Beitollahi H. (2019). A new electrochemical DNA biosensor based on modified carbon paste electrode using graphene quantum dots and ionic liquid for determination of topotecan. Microchem. J..

[34] Mohanraj J., Durgalakshmi D., Rakkesh R.A., Balakumar S., Rajendran S., Karimi-Maleh H. (2020). Facile synthesis of paper based graphene electrodes for point of care devices: A double stranded DNA (dsDNA) biosensor. J. Colloid Interface Sci..

[35] Fornari F.A., Randolph J.K., Yalowich J.C., Ritke M.K., Gewirtz D.A. (1994). Interference by doxorubicin with DNA unwinding in MCF-7 breast tumor cells. Mol. Pharmacol..

[36] Jawad B., Poudel L., Podgornik R., Ching W.-Y. (2020). Thermodynamic Dissection of the Intercalation Binding Process of Doxorubicin to dsDNA with Implications of Ionic and Solvent Effects. J. Phys. Chem. B.

[37] Suganya S., Kumar P.S., Saravanan A. (2017). Construction of active bio-nanocomposite by inseminated metal nanoparticles onto activated carbon: Probing to antimicrobial activity. IET Nanobiotechnol..

[38] Karaman C. (2021). Orange Peel Derived-Nitrogen and Sulfur Co-doped Carbon Dots: A Nano-booster for Enhancing ORR Electrocatalytic Performance of 3D Graphene Networks. Electroanalysis.

[39] Akça A., Karaman O., Karaman C. (2021). Mechanistic Insights into Catalytic Reduction of N2O by CO over Cu-Embedded Graphene: A Density Functional Theory Perspective. ECS J. Solid State Sci. Technol..

[40] Karaman C., Karaman O., Atar N., Yola M.L. (2021). Tailoring of cobalt phosphide anchored nitrogen and sulfur co-doped three dimensional graphene hybrid: Boosted electrocatalytic performance towards hydrogen evolution reaction. Electrochim. Acta.

[41] Ahmadi H., Hosseini E., Cha-Umpong W., Abdollahzadeh M., Korayem A.H., Razmjou A., Chen V., Asadnia M. (2020). Incorporation of Natural Lithium-Ion Trappers into Graphene Oxide Nanosheets. Adv. Mater. Technol..

[42] Cha-Umpong W., Hosseini E., Razmjou A., Zakertabrizi M., Korayem A.H., Chen V. (2019). New molecular understanding of hydrated ion trapping mechanism during thermally-driven desalination by pervaporation using GO membrane. J. Membr. Sci..

[43] Karimi-Maleh H., Ranjbari S., Tanhaei B., Ayati A., Orooji Y., Alizadeh M., Karimi F., Salmanpour S., Rouhi J., Sillanpää M. (2021). Novel 1-butyl-3-methylimidazolium bromide impregnated chitosan hydrogel beads nanostructure as an efficient nanobio-adsorbent for cationic dye removal: Kinetic study. Environ. Res..

[44] Karaman C., Karaman O., Atar N., Yola M.L. (2021). Sustainable Electrode Material for High-Energy Supercapacitor: Biomass-Derived Graphene-Like Porous Carbon with Three Dimensional Hierarchically Ordered Ion Highways. Phys. Chem. Chem. Phys..

[45] Cha-umpong W., Li Q., Razmjou A., Chen V. (2021). Concentrating brine for lithium recovery using GO composite pervaporation membranes. Desalination.

[46] Mohammad M., Lisiecki M., Liang K., Razmjou A., Chen V. (2020). Metal-Phenolic network and metal-organic framework composite membrane for lithium ion extraction. Appl. Mater. Today.

[47] Razmjou A., Asadnia M., Hosseini E., Korayem A.H., Chen V. (2019). Design principles of ion selective nanostructured membranes for the extraction of lithium ions. Nat. Commun..

[48] Mirzajanzadeh M., Tabatabaei M., Ardjmand M., Rashidi A., Ghobadian B., Barkhi M., Pazouki M. (2015). A novel soluble nano-catalysts in diesel–biodiesel fuel blends to improve diesel engines performance and reduce exhaust emissions. Fuel.

[49] Karaman C., Aktaş Z., Bayram E., Karaman O., Kızıl Ç. (2020). Correlation between the Molecular Structure of Reducing Agent and pH of Graphene Oxide Dispersion on the Formation of 3D-Graphene Networks. ECS J. Solid State Sci. Technol..

[50] Merkoçi A., Pumera M., Llopis X., Pérez B., Del Valle M., Alegret S. (2005). New materials for electrochemical sensing VI: Carbon nanotubes. TrAC Trends Anal. Chem..

[51] Trott O., Olson A.J. (2010). AutoDock Vina: Improving the speed and accuracy of docking with a new scoring function, efficient optimization, and multithreading. J. Comput. Chem..

[52] Morris G.M., Huey R., Lindstrom W., Sanner M.F., Belew R.K., Goodsell D.S., Olson A.J. (2009). AutoDock4 and AutoDockTools4: Automated docking with selective receptor flexibility. J. Comput. Chem..

[53] Berman H.M., Battistuz T., Bhat T.N., Bluhm W.F., Bourne P.E., Burkhardt K., Feng Z., Gilliland G.L., Iype L., Jain S. (2002). The protein data bank. Acta Crystallogr. Sect. D Biol. Crystallogr..

[54] Weiner S.J., Kollman P.A., Nguyen D.T., Case D.A. (1986). An all atom force field for simulations of proteins and nucleic acids. J. Comput. Chem..

[55] Gasteiger J., Marsili M. (1980). Iterative partial equalization of orbital electronegativity—a rapid access to atomic charges. Tetrahedron.

[56] Delano W.L. (2002). The PyMOL Molecular Graphics System, Version 1.8.

[57] Hashemzadeh N., Hasanzadeh M., Shadjou N., Eivazi-Ziaei J., Khoubnasabjafari M., Jouyban A. (2016). Graphene quantum dot modified glassy carbon electrode for the determination of doxorubicin hydrochloride in human plasma. J. Pharm. Anal..

[58] Hahn Y., Lee H.Y. (2004). Electrochemical behavior and square wave voltammetric determination of doxorubicin hydrochloride. Arch. Pharmacal Res..

[59] Guo Y., Chen Y., Zhao Q., Shuang S., Dong C. (2011). Electrochemical sensor for ultrasensitive determination of doxorubicin and methotrexate based on cyclodextrin-graphene hybrid nanosheets. Electroanalysis.

[60] Rahimi M., Gh A.B., Fatemi S.J. (2019). A new sensor consisting of bird nest-like nanostructured nickel cobaltite as the sensing element for electrochemical determination of doxorubicin. J. Electroanal. Chem..

